# Exploration of lymphoproliferative disease virus strains in wild turkeys (*Meleagris gallopavo*) in Iowa, USA

**DOI:** 10.1371/journal.pone.0361804

**Published:** 2026-11-02

**Authors:** Kelsey C. Smith, David Navarro, Julie A. Blanchong

**Affiliations:** Department of Natural Resource Ecology and Management, Iowa State University, Ames, Iowa, United States of America; Cornell University, UNITED STATES OF AMERICA

## Abstract

Wild turkeys (*Meleagris gallopavo*) were nearly extirpated in the early 1900’s and extensive restoration efforts utilizing translocated wild turkeys were employed to restore populations. Although these efforts were successful, populations have again been in decline due to unknown causes. Lymphoproliferative disease virus (LPDV) was first detected in the United States in 2009. The coinciding appearance of LDPV and declining populations have led to studies in several states and provinces in North America to characterize its prevalence and distribution due to concerns about its potential role in population declines. The spread of disease agents is a common outcome during translocation programs and could be related to the spread of LPDV. Prior studies have performed phylogenetic analyses to examine genetic variation across and geographic clustering among strains. In this study we aimed to identify the diversity of LPDV strains present in wild turkeys in Iowa and if there was geographic clustering within the state or with other locations where LPDV has been detected. We sequenced a subset (n = 77) of hunter-harvested samples covering eight of the nine agricultural regions in Iowa that tested PCR positive with the viral *gag* gene. We performed phylogenetic analysis on 73 unique Iowan strains as well as previously catalogued strains from other regions retrieved from the National Center for Biotechnology Information (NCBI) GenBank database. Overall, Iowa strains did not show geographic clustering within the state or with any other specific geographic regions. Our study demonstrated that there is a high level of genetic diversity among LPDV strains in Iowa and did not provide evidence of connection with a particular location external to Iowa as a source of introduction of LPDV to the state.

## Introduction

Wild turkeys (*Meleagris gallopavo silvestris*) have served as an important source of game in North America, both currently [1] and historically [2]. Prior to European settlement, wild turkeys were abundant in North America, occurring in 39 of the continental states [3,4]. However, they were nearly extirpated in the early 1900’s due to settlement and a lack of game laws and by 1920, were found in only 18 states [2]. State led restoration programs were soon developed and attempted to restore turkey populations through the translocation of wild-trapped birds. These efforts were largely successful and resulted in turkeys found in all 48 continental states [3,5]. Within 50 years, turkey populations grew from near extirpation to 5.6 million [5]. Historically, Iowa has seen similar trends as the rest of North America, with Eastern wild turkeys from Missouri successfully relocated into Lee County in 1965–1966 and again in 1968 in Lucas County [6–8]. By 1971, turkey populations in Iowa were sufficient for trap-and-transfer to other counties within the state. Since the initial release, 3,578 birds from Missouri and re-established Iowa populations have been relocated to 259 sites in Iowa [8].

Although initial restoration was successful, in the early 2000’s many states again began to notice declines in turkey populations [9,10]. Declines in spring harvest have been reported in 30 states, and fall turkey harvest in 23 states, including Iowa [10]. While the cause of declines is currently unknown, concerns about disease impacts have been increasing [11–14]. The first documentation of lymphoproliferative disease virus (LPDV) within the United States in 2009 has raised questions as to its potential role in declines [15].

LPDV is an oncogenic exogenous avian retrovirus first identified in domestic turkeys in Europe and Israel during the 1970’s [16]. Although domestic turkeys experienced increased morbidity and mortality [17], infection in hunter-harvested wild turkeys appears to more often be asymptomatic though not in all cases [16,18]. If present, clinical symptoms tend to be non-specific [17]. Little is currently known about how natural infection is transmitted between individuals, although it has experimentally been demonstrated to occur horizontally [16].

LPDV appears to be geographically widespread and has been identified in U.S. states ranging down the East coast and as far West as Colorado [11–14,16,19–22], as well as in three Canadian provinces: Manitoba, Ontario, and Quebec [23,24]. The origin of LPDV is currently unknown, although because wild turkeys are not migratory and this virus is widespread, it is thought to potentially be endemic in the United States [16] and likely present prior to 2009 [18,24]. The fact that turkeys are native only to North America and lack of recent detections in Israel and Europe provide evidence suggesting it may have been brought over to Europe and the Middle East with infected birds [16,19]. Domesticated turkeys (originating from Mexico) were initially introduced to Europe through Spain in the early 16th century and records indicate wild birds were also shipped to Europe from Virginia [25].

Pathogens are a common concern in translocation programs, whether introduced to a “new” habitat from introduced individuals or posing a threat to immunologically naïve wildlife relocated to a target site where a pathogen is already present [26–28]. While the potential role of past translocations in LPDV distribution is unclear, the spread of pathogens due to relocating wildlife is not a novel occurrence, and some studies have found evidence that might suggest a relationship between LPDV and historic North American wild turkey restoration efforts [24,29].

An initial phylogenetic analysis of LPDV in wild turkeys identified two major clades: the Israeli prototype and North American [16]. Thus far, in North America only four wild turkeys from South Carolina clustered closely with the Israeli prototype strain, indicating intercontinental spread. Phylogenetic analysis of LPDV in New York showed samples were divided into 28 distinct clades, all falling into the larger North American clade [29]. Spatial patterns among viral strains and clades with a common translocation origin were identified, indicating population restoration efforts may have played a role in the spread of LPDV. Thomas et al. [19] found evidence of geographic clustering, although the small number of sequences from individual states hindered the strength of analysis. A study in Canada found viral strains in Quebec to be clustered with strains detected in Ontario, Indiana, Pennsylvania, New York, New Hampshire, and Maine, while strains identified in Manitoba were more closely related to strains found in South Carolina, North Carolina, and Missouri [24]. This similarity in viral sequences among birds that would otherwise be geographically isolated might suggest that LPDV was spread through introduced birds rather than natural movement between adjacent populations. Similar to the US, parts of Canada, including Quebec and Manitoba, also re-established extirpated populations through translocations. Infected turkeys from Ontario clustered with samples from Quebec [24] but also exhibited high nucleotide identity (high level of similarity when comparing nucleotide bases present in the viral genome with those of another sequence) with sequences from New York, New Jersey, and Pennsylvania [23].

In this study our objective was to identify the diversity of LPDV strains present in wild turkeys in the midwestern US state of Iowa and to determine if there was geographic clustering within the state or with other locations where LPDV has been detected.

## Materials and methods

### Sample collection

In collaboration with the Iowa Department of Natural Resources (IADNR), we collected the tarsi of hunter-harvested wild turkeys between 2019 and 2021 (n = 1022). The location (coordinates, road intersection, township/range/section, or township/range) and county of harvest were reported for each bird. Seven tarsi from road-killed turkeys and samples provided by the IADNR State Wildlife Veterinarian (submitted for necropsy) were also included. We stored samples in a -20^o^C or -80^o^C freezer upon receipt. Because samples were collected from hunter-harvested or road-killed animals the Iowa State University Institutional Animal Care and Use Committee (IACUC) did not require an IACUC protocol for this study. All samples from hunter-harvested birds were submitted by hunters with licenses from the IADNR on private or public land. None of the authors participated in hunting the turkeys.

### Bone marrow and DNA extraction

We removed bone marrow from bones using forceps and probes, cleaning instruments with a 10% bleach solution and sterilizing with ethanol and flame between each use to prevent cross-contamination. We aliquoted approximately 25 mg of tissue into a 1.5mL microcentrifuge tube and refrigerated the tissue until DNA extraction. We froze surplus bone marrow at -20^o^C or -80^o^C in a separate tube.

We performed DNA extraction using the DNeasy Blood & Tissue Kit (Qiagen) following the manufacturer’s protocol with one adjustment: the volume of AE buffer used during elution was decreased from 200 µL to 100 µL to increase final DNA concentration. This was again decreased to 50 µL for samples with less than 25 mg of bone marrow. We quantified the concentration of extracted DNA with a Denovix DS-11 spectrophotometer (NanoDrop Technologies). We created standardized working samples by diluting with sterile water to a concentration of 50ng/µL to ensure consistency during PCR. We left samples undiluted if their concentration was less than 50ng/µL.

### PCR

We tested samples for LPDV by conducting PCR using primers developed by Allison et al. [16] targeting the P31 and a part of the CA (capsid) domains of the virus’s *gag* gene. We performed PCR using the protocol described by Alger et al. [30] with minor adjustments. Specifically, we amplified products with a Mastercycler EP Gradient (Eppendorf) in a 20 µL reaction consisting of 10 µL HotStarTaq Plus Master Mix (Qiagen), 1 µL of each 10µM primer, 6 µL water, and 2 µL DNA. Reaction conditions included a denaturation step at 95^o^C for 5 min, followed by denaturation at 95^o^C for 30 sec, annealing at 54^o^C for 30 sec, and extension at 72^o^C for 1 min for 34 cycles, ending with extension at 72^o^C for 10 min.

We included negative and positive controls in each reaction. The negative control was comprised of the sample reaction but substituted DNA with an additional 2 µL of water. Positive controls, provided by R. Renshaw at Cornell University, were spleen/liver and undefined tissues from New York turkeys that submitted to Cornell University for necropsy because they showed signs of disease and were confirmed LPDV positive through pathology followed by sequencing. We supplemented positive controls by purifying a subset of positive samples to isolate DNA with the QIAquick PCR Purification Kit (Qiagen). We sequenced four of the additional positive controls for confirmation of LPDV infection. We visualized PCR results using agarose gel electrophoresis. LPDV infection was indicated by the presence of a band matching that of the positive control.

### Sequencing

Sequencing was performed using samples that were LPDV positive on agarose gels using the *gag* gene so that results were comparable to other studies which have also used the *gag* gene. Of the total samples, 153 tested positive for LPDV using the *gag* gene (additional details about these samples in S1 and [31]). Of these samples, we selected a subset from each county, when available, to examine genetic variation across Iowa (n = 77) (S1 Appendix). We purified PCR products using ExoSAP-IT Express PCR Product Cleanup Reagent (Thermo Fisher Scientific) following the manufacturer’s protocol. We quantified DNA concentration of purified samples using an Invitrogen Qubit 4 Fluorometer (Thermo Fisher Scientific). We created a 10 µL working sample with a concentration of 10ng/µL by diluting with sterile water. We submitted samples with a lower quantity or concentration for sequencing without dilution.

We performed Sanger sequencing [32] using the forward *gag* primer at the Iowa State University DNA Facility with an Applied Biosystems 3730xl DNA Analyzer (Thermo Fisher Scientific). We confirmed sequences to be LPDV by conducting a National Center for Biotechnology Information (NCBI) GenBank BLAST search. We used Geneious Prime version 2021.1.1 to align Iowa sequences with a suite of five reference sequences (previously catalogued sequences: accession numbers KC801996, KC801997, KC801999, KC802000, KC802001) that were composed of varied nucleotide sequences to check base calls. We marked ambiguous nucleotide sites with the corresponding International Union of Pure and Applied Chemistry (IUPAC) code when two peaks of roughly equal height at the same base position were present and could not be resolved: A and G = R, C and T = Y, A and T = W, G and T = K. We deposited sequences in GenBank under accessions OR026188- OR026260.

### Phylogenetic analysis

We performed phylogenetic analysis on partial p31/capsid (CA) LPDV sequences identified in Iowa wild turkeys to examine relationships between strains within Iowa and with previously catalogued sequences from other studies in GenBank. We trimmed our Iowa sample sequences to an equal length of 406 base pairs. We trimmed sequences so that genetic diversity was a result of different nucleotide sequences rather than differences in the number of bases available. We used MEGA version 7.0.26 to construct a maximum likelihood phylogenetic tree of Iowa samples using a general time-reversible and discrete gamma (GTR + G) model of nucleotide substitution (5 categories, estimated gamma shape parameter of 0.3439) with Nearest-Neighbor Interchange (NNI) branch-swapping. We inferred support for individual nodes using a bootstrap analysis with 500 replicates. We selected a GTR + G model to maintain consistency with other studies [16,19,23,24,29], because it was found to have a better fit than other models describing LPDV [33], and because of the high mutation rate of viruses [34]. Studies have shown that viruses can have different substitution rates based off of both base identity (e.g., A versus G nucleotides) and site position within the genome, and phylogenetic trees based on models accounting for these differences have been shown to provide a better fit [34,35]. GTR models account for varying base frequencies and unequal probabilities of nucleotide substitutions with respect to the base identity [36]. Gamma distributions account for heterogeneity in substitution rates among different positions along the sequence [36]. To visualize whether strains clustered spatially, we color-coded samples according to the agricultural region they were harvested in. We chose agricultural region because sample sizes at the county level were often small. Iowa is split into nine different agricultural regions, each encompassing between nine and twelve counties, allowing us to examine spatial variation in LDPV strains.

To examine relationships between LPDV strains in Iowa and those identified in previous studies, we conducted an additional phylogenetic analysis using a combination of Iowa samples and all available sequences that were included in the NCBI GenBank database from other studies in other regions at the time of this study (n = 347, specific details below). We retrieved sequences from other studies from the NCBI GenBank database. We used MUSCLE to align sequences [37] and trim these sequences to 339 bases. We trimmed Iowa strains down from 406 base pairs to maximize the number of strains included from other studies, some of which had shorter sequences. We used the same phylogenetic analysis methods described above with one exception: the estimated gamma shape parameter was 0.5478. We designated the outgroup based on a prior study [16] and included the Israeli strain (GenBank accession U09568) and four isolates from South Carolina (GenBank accessions KC801975, KC801976, KC801991, KC802005). Ingroup sequences included 1 sample from Arizona, 1 from Colorado, 5 from Florida, 12 from Georgia, 1 from Indiana, 5 from Kansas, 26 from Louisiana, 3 from Massachusetts, 1 from Maryland, 19 from Maine, 8 from Missouri, 4 from Mississippi, 3 from North Carolina, 16 from New Hampshire, 6 from New Jersey, 161 from New York, 1 from Ohio, 10 from Ontario, 4 from Pennsylvania, 14 from South Carolina, 9 from Texas, 6 from Virginia, 4 from Vermont, and 22 from West Virginia [S2 Appendix,16,19,23,29,33]. At the time of this analysis, there were additional LPDV sequences from Manitoba, Quebec, and one sample from Texas in the GenBank database, but we excluded these from analysis because they would have resulted in further trimming of sequences. We collapsed strains on the phylogenetic tree into what visually appeared to be distinct, closely related groups to best visualize where Iowa strains were on the tree relative to the placement of those identified in other states and provinces.

## Results

We sequenced LPDV positive samples (n = 77) from a total of 46 out of 57 Iowa counties where LPDV was detected with the *gag* gene, covering eight of the nine agricultural regions [S1 Appendix, Table 1]. Counties with positive samples that were unrepresented were due to a sample concentration that was too low for successful sequencing. The length of sequences ranged from 400 to 413 bases prior to trimming to an equal length. We identified ambiguous bases in 14 sequences: 03/IA/2022, 10/IA/2022, 13/IA/2022, 16/IA/2022, 19/IA/2022, 23/IA/2022, 26/IA/2022, 31/IA/2022, 50/IA/2022, 53/IA/2022, 55/IA/2022, 59/IA/2022, 61/IA/2022, 71/IA/2022. LPDV strains identified in Iowa wild turkeys demonstrated a high level of genetic diversity, with only three cases of turkeys with identical LPDV sequences (01/IA/2022 – samples 10 and 326, 04/IA/2022 – samples 26 and 127 and 26, 42/IA/2022 – samples 565 and 643). We excluded one sample because its addition would have resulted in the loss of an additional 6 base pairs, and therefore genetic diversity (27/IA/2022), resulting in 73 unique sequences included in the phylogenetic analysis. Both pairwise identity and percent identical sites of Iowa samples with their closest match in GenBank ranged from 93.4% to 99.0%. Averaged across the 73 unique sequences used in analysis, the nucleotide composition was as follows: T = 18.2%, C = 20.5%, A = 28.9%, and G = 32.4%.

**Table 1:** List of lymphoproliferative disease virus (LPDV) strains (unique *gag* gene sequences) from wild turkeys in each Iowa agricultural region, 2019-2021.

Region	Strains
**Central**	65/IA/2022; 17/IA/2022; 25/IA/2022; 08/IA/2022; 52/IA/2022; 66/IA/2022
**East Central**	19/IA/2022; 33/IA/2022; 10/IA/2022; 26/IA/2022; 59/IA/2022; 20/IA/2022; 23/IA/2022; 04/IA/2022a
**North Central**	62/IA/2022; 64/IA/2022; 46/IA/2022; 54/IA/2022; 32/IA/2022; 73/IA/2022; 60/IA/2022; 58/IA/2022; 29/IA/2022; 56/IA/2022; 42/IA/2022a
**Northeast**	31/IA/2022; 44/IA/2022; 48/IA/2022; 41/IA/2022; 72/IA/2022; 06/IA/2022; 28/IA/2022; 15/IA/2022; 53/IA/2022; 04/IA/2022b; 30/IA/2022; 49/IA/2022; 74/IA/2022; 14/IA/2022; 42/IA/2022b; 22/IA/2022
**South Central**	70/IA/2022; 12/IA/2022; 37/IA/2022; 61/IA/2022; 71/IA/2022; 50/IA/2022; 69/IA/2022; 21/IA/2022; 68/IA/2022; 02/IA/2022; 11/IA/2022; 01/IA/2022a; 01/IA/2022b
**Southeast**	35/IA/2022; 43/IA/2022; 18/IA/2022; 36/IA/2022; 07/IA/2022; 34/IA/2022; 40/IA/2022; 13/IA/2022; 51/IA/2022; 38/IA/2022; 57/IA/2022; 03/IA/2022; 16/IA/2022
**Southwest**	05/IA/2022; 63/IA/2022; 09/IA/2022; 39/IA/2022; 45/IA/2022
**West Central**	24/IA/2022; 55/IA/2022; 67/IA/2022; 27/IA/2022; 47/IA/2022

Strain names consist of a strain identification number, state abbreviation, and year (of sequencing). No samples from the Northwest region were sequenced. For additional details see S1 Appendix.

Our phylogenetic analysis of Iowa samples indicated that overall, viral strains were dispersed across agricultural regions rather than grouped together spatially, with the exception of several small groups from the same region (Fig 1). Low bootstrap support values indicated that the majority of groupings were weak, with little confidence for strain groupings. When we compared Iowa samples with samples from other regions, Iowa samples grouped with the majority of North American isolates rather than the Israeli/South Carolina outgroup (Fig 2, S3 Appendix). Iowa strains did not appear to cluster with any specific regions and were instead found to be closely related to strains from many other regions.

**Fig 1:**
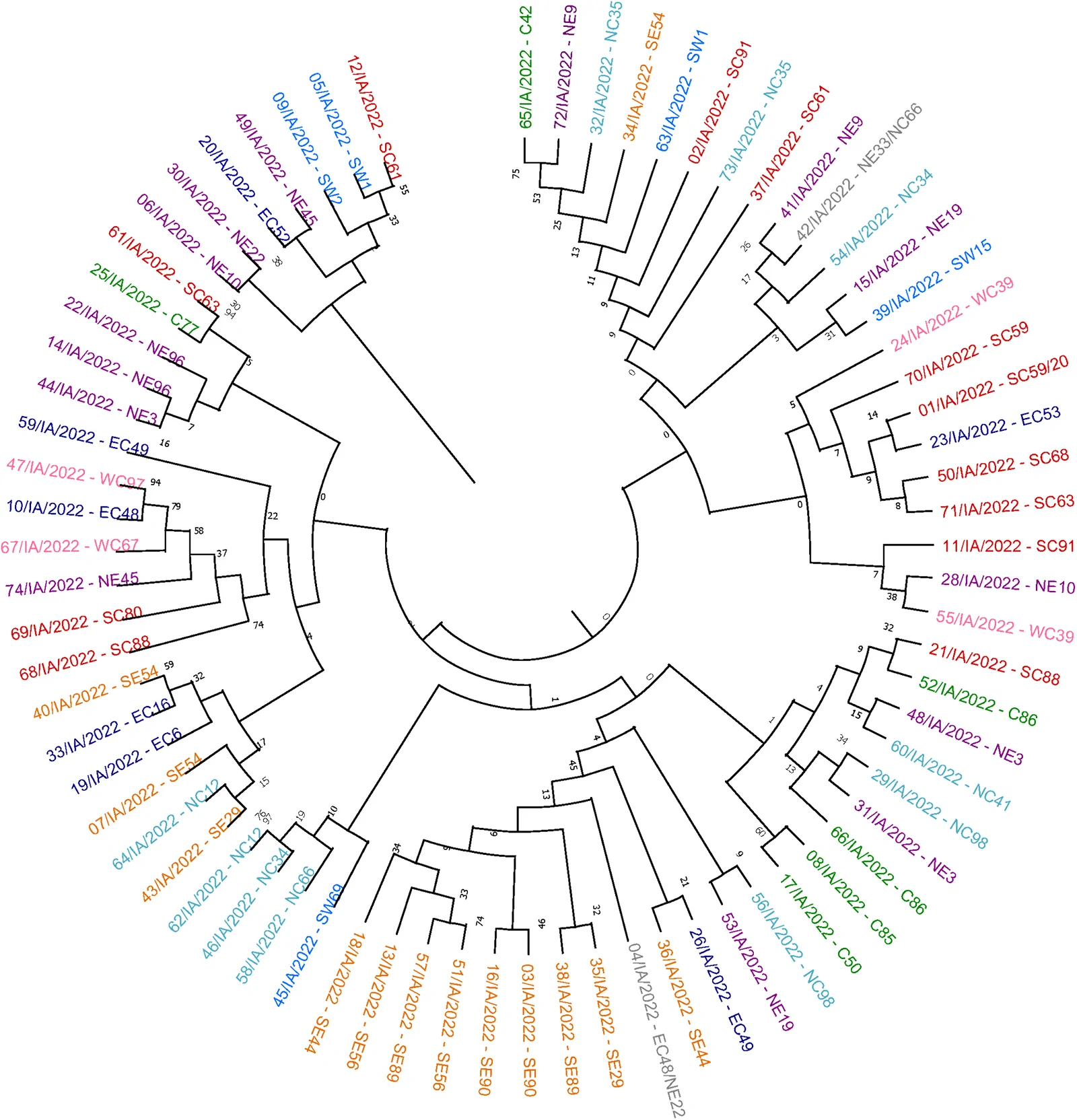
Phylogenetic relationships of lymphoproliferative disease virus (LPDV) strains from Iowa wild turkeys, 2019-2021. Sequences are named with their strain ID, state abbreviation, and year followed by the agricultural region abbreviation and county ID for the location of harvest. Sequences are color coded according to agricultural region (see Table 1, S1). Bootstrap values are indicated.

**Fig 2:**
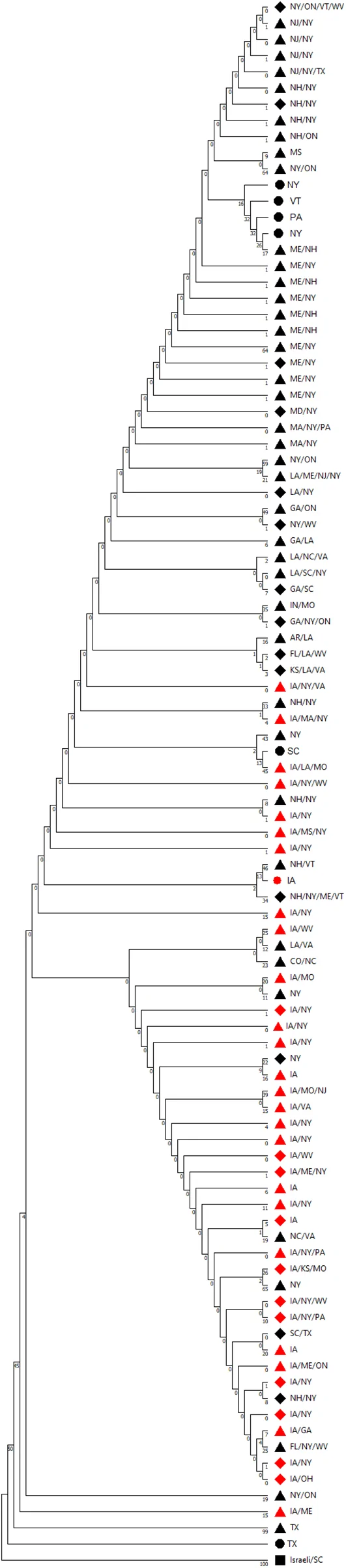
Phylogenetic relationships of lymphoproliferative disease virus (LPDV) strains from Iowa wild turkeys (2019-2021) and catalogued sequences. Groups containing samples from Iowa are bolded and are denoted by a red node marker. The outgroup is designated with a square marker. Node marker shape, with the exception of the outgroup, is based on the number of samples within the group: circle = 1, triangle = 2-4, diamond = 5 + . Bootstrap values are indicated.

## Discussion

We performed our study to explore genetic relationships among LPDV strains found in Iowa wild turkeys within the state as well as with several other states and provinces where LPDV has been detected in wild turkeys. Out of the 77 samples sequenced, we included 73 unique 406 nucleotide sequences in phylogenetic analysis, indicating that there is high genetic diversity of LPDV within Iowa. This is similar to other studies in the Northeast U.S. that examined LPDV on a statewide scale and also found high levels of diversity [12,29]. Iowa strains grouped more closely with other North American strains compared to the Israeli clade, as has been the case in other studies, with the exception of four sequences identified in South Carolina [16,19,23,24,29,33].

With the exception of a few small groups, strains did not appear to display significant amounts of geographical clustering within Iowa, with strains widely dispersed across eight large agricultural regions [Fig 1]. A study in Maine also found little apparent geographic clustering among strains with similar low branch support for genetic lineages [33]. If historic translocations played a role in the dispersal of LPDV one might expect strains would be clustered spatially, which was not observed for these data. However, some translocated populations in Iowa came from other established Iowa populations, so it is possible that if translocations did play a role, their impact was masked by the movement of populations within the state. Specifically, if infected birds were brought into the state, successfully established, then were used for further translocations around the state, there would likely be no noticeable spatial patterns. Another possibility for the lack of geographic clustering is the manner in which regions were defined. We chose agricultural regions for the purpose of achieving good spatial distribution of samples across the state, and they may not be a unit that is biologically relevant to wild turkeys. Looking at relationships among sequences with respect to population ranges, natural dispersal patterns, or finer-scale translocation sites might reveal more patterns.

Our phylogenetic analysis examining the relationship between Iowa strains and other geographic areas where LPDV has been detected did not identify clustering with any specific areas. We found Iowa strains were closely related to strains from a wide range of North American regions: Northeast, Midwest, Southeast, Mid-Atlantic, and Canada [Fig 2, S3 Appendix]. Some studies have found evidence of geographic clustering by state but have noted that there were small sample sizes and weak support for groupings, making true relationships difficult to define [16,19]. A study conducted in Canada found some of their sequences clustered with states that were not close in proximity, which may be indicative of the role of translocations in the spread of LPDV [24]. A New York study found associations between strongly supported clades that were explainable either by geographic proximity, common sources of translocation, or a combination of both [29]. In addition to translocation of turkeys to Iowa for the purposes of restoration, Iowa also exports birds to other states. While translocation records for turkeys sent from Iowa to other states are incomplete, birds have been sent to South Dakota and Texas [38,39]. At the time of this study, no LPDV sequences had been submitted to GenBank from South Dakota, thus no inferences can be drawn. Three Iowa LPDV strain samples grouped somewhat closely with some strains from Texas raising the possibility of a link associated with translocations.

We used relatively few samples (one to three per county) for analyses. An increase in sample size may have improved the accuracy of inferred genetic relationships among strains and resulted in a phylogenetic tree with more branch support. Another factor which may have impacted results is that we left samples with ambiguities in the analysis when another study excluded them [33]. Ambiguities occur when two peaks of roughly equal height at the same base position were present and could not be resolved. Ambiguities may have been a result of machine error during sequencing, but it is also possible they were a result of two strains occurring at different locations within the turkey’s genome where the strains possess different nucleotides at the same base site [33]. Although not demonstrated in wild birds, a study in domestic turkeys identified three LPDV proviral clones originated from multiple sites within a turkey genome [40].

Another limitation of our and other phylogenetic analyses of LPDV to date is that the entire viral genome was not sequenced. By only examining a small portion of the genome, genetic diversity may be inaccurately estimated and relationships among strains may not be accurately characterized. Mutation rates in viruses can vary with nucleotide site position [35]. A study found that, though similar, the four LPDV genes (*gag*, *pro*, *pol*, *env*) had varying levels of nucleotide conservation [16].

The wide diversity, low branch support, and lack of geographical clustering we identified in Iowa LPDV strains supports conclusions drawn by other studies of viral spread resulting from turkey populations that are highly connected, both artificially and naturally [12,16,21,22]. The role of translocations in the dispersal of LPDV has not been definitively demonstrated, but LPDV’s potential association with morbidity and mortality that might be associated with observed population declines indicates continued research is warranted and the potential for disease spread should be taken into consideration if wild turkeys continue to be translocated.

## Supporting information

S1 Appendix Lymphoproliferative disease virus strain ID for 77 Iowa wild turkeys sampled from 2019–2021. (DOCX)

S2 Appendix Previously cataloged LPDV strains used in phylogenetic analysis. (DOCX)

S3 Appendix Phylogenetic tree showing individual samples summarized in Fig 2. (DOCX)

## References

[1] Chapagain BP, Poudyal NC, Joshi O, Watkins C, Applegate RD. Seasonal and regional differences in economic benefits of turkey hunting. Wildl Soc Bull. 2020;44(2):271–80. doi: 10.1002/wsb.1093

[2] Hughes TW, Lee K. The role of recreational hunting in the recovery and conservation of the wild turkey (*Meleagris gallopavo* spp.) in North America. Int J Environ Stud. 2015;72(5):797–809. doi: 10.1080/00207233.2015.1022998

[3] Mitchell MD, Kimmel RO, Snyders J. Reintroduction and range expansion of Eastern wild turkeys in Minnesota. Geographical Review. 2011;101(2):269–84. doi: 10.1111/j.1931-0846.2011.00091.x

[4] Kennamer JE, Kennamer MC, Brenneman R. History of the wild turkey in North America. National Wild Turkey Federation Wildl Bull. 2015;15:8.

[5] Bromley PT. Lessons from the wild turkey. Biologist (London). 2001;48(6):292. 11740082

[6] Little T. Wild turkey restoration in “marginal” Iowa habitats. In: Fourth National Wild Turkey Symposium. Little Rock, Arkansas, 1980.

[7] Dinsmore JJ. Wild turkeys. In: A country so full of game: the story of wildlife in Iowa. Iowa City: University of Iowa Press; 1994. 115–20.

[8] Coffey J. Wild turkeys. Trends in Iowa wildlife populations and harvest 2017-2018. 2018. 35–61.

[9] Casalena MJ, Bowling AC, Schiavone MV, Gregg ID, Brown J. Understanding the new normal: Wild turkeys in a changing northeastern landscape. In: Miller DA, editor. Proceedings of the eleventh national wild turkey symposium. Tucson, Arizona: The National Wild Turkey Federation; 2015. 45–56.

[10] Eriksen RE, Brown TA, Scott KB, Hughes TW, Akridge MD, Penner CS. Status and distribution of wild turkeys in the United States: 2014 status. In: Miller DA, editor. Proceedings of the eleventh national wild turkey symposium. Tucson, Arizona: The National Wild Turkey Federation; 2016. 7–18.

[11] MacDonald AM, Johnson JB, Casalena MJ, Nemeth NM, Kunkel M, Blake M. Active and passive disease surveillance in wild turkeys (*Meleagris gallopavo*) from 2008-2018 in Pennsylvania, USA. Wildl Soc Bull. 2021;46(e1289). doi: org/10.1002/wsb.1289

[12] Shea SA, Gonnerman M, Blomberg E, Sullivan K, Milligan P, Kamath PL. Pathogen survey and predictors of lymphoproliferative disease virus infection in wild turkeys (*Meleagris gallopavo*). J Wildl Dis. 2022;58(3):537–49. doi: 10.7589/JWD-D-21-00152 35704504

[13] Thiemann R, Dalton MF, Rose H, Baughman B, Butler A, Adcock K, et al. An investigation of the cause of wild turkey mortality in Mississippi. Avian Dis. 2022;66(2):237–42. doi: 10.1637/aviandiseases-D-22-00007 35838749

[14] Haynes E, Yabsley MJ, Nemeth NM, Danks ZD, Stasiak I, Garrett KB, et al. Health assessment of adult male eastern wild turkeys (*Meleagris gallopavo* silvestris) from Western Kentucky, USA. J Wildl Dis. 2024;60(3):660–9. doi: 10.7589/JWD-D-23-00162 38584308

[15] Alger K, Bunting E, Schuler K, Whipps CM. Risk factors for and spatial distribution of lymphoproliferative disease virus (lpdv) in wild turkeys (*Meleagris gallopavo*) in New York state, USA. J Wildl Dis. 2017;53(3):499–508. doi: 10.7589/2016-06-137 28328350

[16] Allison AB, Kevin Keel M, Philips JE, Cartoceti AN, Munk BA, Nemeth NM, et al. Avian oncogenesis induced by lymphoproliferative disease virus: A neglected or emerging retroviral pathogen?. Virology. 2014;450–451:2–12. doi: 10.1016/j.virol.2013.11.037 24503062 PMC3925403

[17] Biggs PM, McDougall JS, Frazier JA, Milne BS. Lymphoproliferative disease of turkeys 1. Clinical aspects. Avian Pathol. 1978;7(1):131–9. doi: 10.1080/03079457808418265 18770365

[18] Niedringhaus KD, Nemeth NM, Sellers HS, Brown JD, Fenton HMA. Multicentric round cell neoplasms and their viral associations in wild turkeys (*Meleagris gallopavo*) in the Southeastern United States. Vet Pathol. 2019;56(6):915–20. doi: 10.1177/0300985819864306 31345138

[19] Thomas JM, Allison AB, Holmes EC, Phillips JE, Bunting EM, Yabsley MJ, et al. Molecular surveillance for lymphoproliferative disease virus in wild turkeys (*Meleagris gallopavo*) from the Eastern United States. PLoS One. 2015;10(4):e0122644. doi: 10.1371/journal.pone.0122644 25897755 PMC4405500

[20] Kreh CD, Palamar MB. Prevalence of lymphoproliferative disease virus in wild turkeys (*Meleagris gallopavo*) in North Carolina. Wildl Soc Bull. 2021;46:e1263. doi: 10.1002/wsb.1263

[21] Cox F, Hardin J, Dittmar R, Edwards D. Molecular surveillance for lymphoproliferative disease virus and reticuloendotheliosis virus in rio grande wild turkeys (*Meleagris gallopavo intermedia*) in Texas, USA. J Wildl Dis. 2022;58(4):909–13. doi: 10.7589/JWD-D-22-00023 36305745

[22] Adcock KG, Berghaus RD, Goodwin CC, Ruder MG, Yabsley MJ, Mead DG, et al. Lymphoproliferative disease virus and reticuloendotheliosis virus detection and disease in wild turkeys (*Meleagris gallopavo*). J Wildl Dis. 2024;60(1):139–50. doi: 10.7589/JWD-D-23-00012 37972643

[23] MacDonald AM, Jardine CM, Bowman J, Susta L, Nemeth NM. Detection of lymphoproliferative disease virus in Canada in a survey for viruses in ontario wild turkeys (*Meleagris gallopavo*). J Wildl Dis. 2019;55(1):113–22. doi: 10.7589/2018-01-013 30124393

[24] MacDonald AM, Barta JR, McKay M, Lair S, Le Net R, Baldwin F, et al. Lymphoproliferative disease virus in Wild Turkeys (*Meleagris gallopavo*) from Manitoba and Quebec, Canada. Avian Dis. 2019;63(3):506–10. doi: 10.1637/aviandiseases-D-19-00102 31967435

[25] Wunz GA. Wild turkeys outside their historic range. In: Dickson JG, editor. The wild turkey: Biology and management. Harrisburg, PA: Stackpole Books; 1992. 361–84.

[26] Cunningham AA. Disease risks of wildlife translocations. Conserv Biol. 1996;10(2):349–53. doi: 10.1046/j.1523-1739.1996.10020349.x

[27] Sainsbury AW, Vaughan-Higgins RJ. Analyzing disease risks associated with translocations. Conserv Biol. 2012;26(3):442–52. doi: 10.1111/j.1523-1739.2012.01839.x 22533691

[28] Lewis J, Tomlinson A, Gilbert M, Alshinetski M, Arzhanova T, Goncharuk M, et al. Assessing the health risks of reintroduction: The example of the Amur leopard, *Panthera pardus orientalis*. Transbound Emerg Dis. 2020;67(3):1177–88. doi: 10.1111/tbed.13449 31833654

[29] Alger K. Lymphoproliferative disease virus (LPDV) in wild turkeys (*Meleagris gallopavo*) in New York State: Diagnostic methods, prevalence, and spatial distribution of a newly discovered pathogen. State University of New York; 2015. https://experts.esf.edu/esploro/outputs/graduate/Lymphoproliferative-Disease-Virus-LPDV-in-Wild/99872807504826

[30] Alger K, Bunting E, Schuler K, Jagne J, Whipps CM. Diagnosing lymphoproliferative disease virus in live wild turkeys (*Meleagris gallopavo*) using whole blood. J Zoo Wildl Med. 2015;46(4):806–14. doi: 10.1638/2015-0037.1 26667537

[31] Smith KC, Blanchong JA. Detection of lymphoproliferative disease virus in Iowa Wild Turkeys (*Meleagris gallopavo*): Comparison of two sections of the proviral genome. PLoS One. 2024;19(2):e0296856. doi: 10.1371/journal.pone.0296856 38346036 PMC10861079

[32] Sanger F, Nicklen S, Coulson AR. DNA sequencing with chain-terminating inhibitors. Proc Natl Acad Sci U S A. 1977;74(12):5463–7. doi: 10.1073/pnas.74.12.5463 271968 PMC431765

[33] Shea SA. Retroviral infection dynamics in Maine’s wild turkeys. University of Maine; 2021. https://maine.primo.exlibrisgroup.com/nde/collectionDiscovery?collectionId=81521275480008681&vid=01MAINE_INST:DigCol&lang=en

[34] Leitner T, Kumar S, Albert J. Tempo and mode of nucleotide substitutions in gag and env gene fragments in human immunodeficiency virus type 1 populations with a known transmission history. J Virol. 1997;71(6):4761–70. doi: 10.1128/JVI.71.6.4761-4770.1997 9151870 PMC191698

[35] Sanjuán R, Domingo-Calap P. Mechanisms of viral mutation. Cell Mol Life Sci. 2016;73(23):4433–48. doi: 10.1007/s00018-016-2299-6 27392606 PMC5075021

[36] Strimmer K, Von Haeseler A. Genetic distances and nucleotide substitution models. In: Salemi M, Vandamme AM, Lemey P, editors. The phylogenetic handbook: A practical approach to phylogenetic analysis and hypothesis testing. 2 ed. Cambridge, UK; New York: Cambridge University Press; 2009. 111–41.

[37] Edgar RC. MUSCLE: a multiple sequence alignment method with reduced time and space complexity. BMC Bioinform. 2004;5:113. doi: 10.1186/1471-2105-5-113 15318951 PMC517706

[38] Shields RD, Flake LD. Survival and reproduction of translocated eastern wild turkeys in a sparsely wooded landscape in Northeastern South dakota. Western North American Naturalist. 2006;66(3):298–309. doi: 10.3398/1527-0904(2006)66[298:sarote]2.0.co;2

[39] Seidel SA, Comer CE, Conway WC, DeYoung RW, Hardin JB, Calkins GE. Influence of translocations on eastern wild turkey population genetics in Texas. J Wildl Mgmt. 2013;77:1221–31. doi: 10.1002/jwmg.575

[40] Chajut A, Yaniv A, Avivi L, Bar-Am I, Tronick SR, Gazit A. A novel approach for establishing common or random integration loci for retroviral genomes. Nucleic Acids Res. 1991;19(15):4299. doi: 10.1093/nar/19.15.4299 1870986 PMC328585

