# Supplementary material for: Exploration of lymphoproliferative disease virus strains in wild turkeys (Meleagris gallopavo) in Iowa, USA
Source: PLoS One. 2026 Nov 2;21(11):e0361804. doi: 10.1371/journal.pone.0361804 (PMC13726951; doi:10.1371/journal.pone.0361804)
Supplement: Supplementary file 1 [file pone.0361804.s001.docx]

| **Sample ID** | **Strain ID** | **County (ID)** | **Agricultural Region** | **Sample ID** | **Strain ID** | **County (ID)** | **Agricultural Region** |
| --- | --- | --- | --- | --- | --- | --- | --- |
| 10 | 01/IA/2022a | Lucas (59) | South Central | 258 | 20/IA/2022 | Johnson (52) | East Central |
| 26 | 04/IA/2022b | Clayton (22) | East Central | 265 | 21/IA/2022 | Union (88) | South Central |
| 31 | 02/IA/2022 | Warren (91) | South Central | 267 | 22/IA/2022 | Winneshiek (96) | Northeast |
| 73 | 03/IA/2022 | Wapello (90) | Southeast | 326 | 01/IA/2022b | Clarke (20) | South Central |
| 127 | 04/IA/2022a | Iowa (48) | East Central | 330 | 23/IA/2022 | Jones (53) | East Central |
| 131 | 05/IA/2022 | Adair (01) | Southwest | 337 | 24/IA/2022 | Guthrie (39) | West Central |
| 135 | 06/IA/2022 | Buchanan (10) | Northeast | 361 | 25/IA/2022 | Polk (77) | Central |
| 139 | 07/IA/2022 | Keokuk (54) | Southeast | 365 | 26/IA/2022 | Jackson (49) | East Central |
| 169 | 08/IA/2022 | Story (85) | Central | 374 | 27/IA/2022 | Woodbury (97) | West Central |
| 172 | 09/IA/2022 | Adams (02) | Southwest | 377 | 28/IA/2022 | Buchanan (10) | Northeast |
| 182 | 10/IA/2022 | Iowa (48) | East Central | 395 | 29/IA/2022 | Worth (98) | North Central |
| 188 | 11/IA/2022 | Warren (91) | South Central | 398 | 30/IA/2022 | Clayton (22) | Northeast |
| 189 | 12/IA/2022 | Madison (61) | South Central | 427 | 31/IA/2022 | Allamakee (03) | Northeast |
| 208 | 13/IA/2022 | Lee (56) | Southeast | 436 | 32/IA/2022 | Franklin (35) | North Central |
| 209 | 14/IA/2022 | Winneshiek (96) | Northeast | 442 | 33/IA/2022 | Cedar (16) | East Central |
| 223 | 15/IA/2022 | Chickasaw (19) | Northeast | 464 | 34/IA/2022 | Keokuk (54) | Southeast |
| 225 | 16/IA/2022 | Wapello (90) | Southeast | 476 | 35/IA/2022 | Des Moines (29) | Southeast |
| 227 | 17/IA/2022 | Jasper (50) | Central | 503 | 36/IA/2022 | Henry (44) | Southeast |
| 243 | 18/IA/2022 | Henry (44) | Southeast | 505 | 37/IA/2022 | Madison (61) | South Central |
| 245 | 19/IA/2022 | Benton (06) | East Central | 506 | 38/IA/2022 | Van Buren (89) | Southeast |
| 520 | 39/IA/2022 | Cass (15) | Southwest | 786 | 57/IA/2022 | Van Buren (89) | Southeast |
| 533 | 40/IA/2022 | Keokuk (54) | Southeast | 795 | 58/IA/2022 | Mitchell (66) | North Central |
| 545 | 41/IA/2022 | Bremer (09) | Northeast | 797 | 59/IA/2022 | Jackson (49) | East Central |
| 565 | 42/IA/2022a | Fayette (33) | Northeast | 803 | 60/IA/2022 | Hancock (41) | North Central |
| 607 | 43/IA/2022 | Des Moines (29) | Southeast | 831 | 61/IA/2022 | Marion (63) | South Central |
| 614 | 44/IA/2022 | Allamakee (03) | Northeast | 865 | 62/IA/2022 | Butler (12) | North Central |
| 617 | 45/IA/2022 | Montgomery (69) | Southwest | 879 | 63/IA/2022 | Adair (01) | Southwest |
| 643 | 42/IA/2022b | Mitchell (66) | North Central | 884 | 64/IA/2022 | Butler (12) | North Central |
| 654 | 46/IA/2022 | Floyd (34) | North Central | 885 | 65/IA/2022 | Hardin (42) | Central |
| 661 | 47/IA/2022 | Woodbury (97) | West Central | 893 | 66/IA/2022 | Tama (86) | Central |
| 681 | 48/IA/2022 | Allamakee (03) | Northeast | 894 | 67/IA/2022 | Monona (67) | West Central |
| 686 | 49/IA/2022 | Howard (45) | Northeast | 929 | 68/IA/2022 | Union (88) | South Central |
| 706 | 50/IA/2022 | Monroe (68) | South Central | 930 | 69/IA/2022 | Ringgold (80) | South Central |
| 725 | 51/IA/2022 | Lee (56) | Southeast | 936 | 70/IA/2022 | Lucas (59) | South Central |
| 736 | 52/IA/2022 | Tama (86) | Central | 955 | 71/IA/2022 | Marion (63) | South Central |
| 762 | 53/IA/2022 | Chickasaw (19) | Northeast | 979 | 72/IA/2022 | Bremer (09) | Northeast |
| 769 | 54/IA/2022 | Floyd (34) | North Central | 988 | 73/IA/2022 | Franklin (35) | North Central |
| 775 | 55/IA/2022 | Guthrie (39) | West Central | 1017 | 74/IA/2022 | Howard (45) | Northeast |
| 783 | 56/IA/2022 | Worth (98) | North Central |  |  |  |  |

**S1 Appendix**. Sample ID, LPCV strain ID based on sequencing of the P31 and part of the CA (capsid) domains of the virus’s *gag* gene, harvest county, and agricultural region for 77 Iowa wild turkeys.

**Notes:**

1. Sample ID = Unique identifier for lymphoproliferative disease virus-positive wild turkey samples
2. Strain ID interpretation = *Gag* gene sequence/State of Iowa/Year of analysis (74 unique sequences)
   a. Instances where the Strain ID ends with “a” or “b” indicates 2 turkeys were identified with the same strain (strains 1, 4, 42).
   b. As noted in the text, strain 27/IA/2022 (sample ID 374) was not included in the phylogenetic analysis.
