# Supplementary material for: Exploration of lymphoproliferative disease virus strains in wild turkeys (Meleagris gallopavo) in Iowa, USA
Source: PLoS One. 2026 Nov 2;21(11):e0361804. doi: 10.1371/journal.pone.0361804 (PMC13726951; doi:10.1371/journal.pone.0361804)
Supplement: Supplementary file 2 [file pone.0361804.s002.docx]

**S2 Appendix.** Location of origin, accession number in GenBank, and associated reference for lymphoproliferative disease virus (LPDV) strains based on sequencing the P31 and a part of the CA (capsid) domains of the virus’s *gag* gene included in phylogenetic analysis exploring relationships of LDPV strains found in Iowa wild turkeys with previously documented strains.

| **Location** | **Accession Number** | **Reference** | **Location** | **Accession Number** | **Reference** |
| --- | --- | --- | --- | --- | --- |
| Arizona | KC802224 | [1] | Georgia | KP299722 | [2] |
| Colorado | KC801953 | [1] | Georgia | KP299723 | [2] |
| Florida | KP299709 | [2] | Georgia | KC801952 | [1] |
| Florida | KP299710 | [2] | Indiana | KC801979 | [1] |
| Florida | KP299711 | [2] | Israeli | U09568 | [3] |
| Florida | KP299712 | [2] | Kansas | KC801949 | [1] |
| Florida | KP299713 | [2] | Kansas | KP299724 | [2] |
| Georgia | KP299696 | [2] | Kansas | KP299725 | [2] |
| Georgia | KP299714 | [2] | Kansas | KP299726 | [2] |
| Georgia | KP299715 | [2] | Kansas | KP299727 | [2] |
| Georgia | KP299716 | [2] | Louisiana | KP299701 | [2] |
| Georgia | KP299717 | [2] | Louisiana | KP299702 | [2] |
| Georgia | KP299718 | [2] | Louisiana | KP299703 | [2] |
| Georgia | KP299719 | [2] | Louisiana | KP299704 | [2] |
| Georgia | KP299720 | [2] | Louisiana | KP299705 | [2] |
| Georgia | KP299721 | [2] | Louisiana | KP299706 | [2] |
| Louisiana | KP299707 | [2] | Maine | KP299761 | [2] |
| Louisiana | KP299708 | [2] | Maine | KC801999 | [1] |
| Louisiana | KP299768 | [2] | Maine | KC802000 | [1] |
| Louisiana | KP299769 | [2] | Missouri | KC801954 | [1] |
| Louisiana | KP299770 | [2] | Missouri | KC801956 | [1] |
| Louisiana | KP299771 | [2] | Missouri | KP299762 | [2] |
| Louisiana | KP299772 | [2] | Missouri | KP299763 | [2] |
| Louisiana | KP299773 | [2] | Missouri | KP299764 | [2] |
| Louisiana | KP299774 | [2] | Missouri | KP299765 | [2] |
| Louisiana | KP299775 | [2] | Missouri | KP299766 | [2] |
| Louisiana | KP299776 | [2] | Missouri | KP299767 | [2] |
| Louisiana | KP299777 | [2] | Mississippi | KC801963 | [1] |
| Louisiana | KP299778 | [2] | Mississippi | KC801972 | [1] |
| Louisiana | KP299779 | [2] | Mississippi | KC801973 | [1] |
| Louisiana | KP299780 | [2] | Mississippi | KC801974 | [1] |
| Louisiana | KP299781 | [2] | North Carolina | KC801971 | [1] |
| Louisiana | KP299782 | [2] | North Carolina | KC801978 | [1] |
| Louisiana | KP299783 | [2] | North Carolina | KC802002 | [1] |
| Louisiana | KP299784 | [2] | New Hampshire | KC801964 | [1] |
| Louisiana | KC801958 | [1] | New Hampshire | KC801965 | [1] |
| Massachusetts | KP299742 | [2] | New Hampshire | KC801966 | [1] |
| Massachusetts | KP299743 | [2] | New Hampshire | KC801967 | [1] |
| Massachusetts | KP299744 | [2] | New Hampshire | KC801968 | [1] |
| Maryland | KC802001 | [1] | New Hampshire | KC801969 | [1] |
| Maine | KC801960 | [1] | New Hampshire | KP299732 | [2] |
| Maine | KP299697 | [2] | New Hampshire | KP299733 | [2] |
| Maine | KP299698 | [2] | New Hampshire | KP299734 | [2] |
| Maine | KP299699 | [2] | New Hampshire | KP299735 | [2] |
| Maine | KP299700 | [2] | New Hampshire | KP299736 | [2] |
| Maine | KP299750 | [2] | New Hampshire | KP299737 | [2] |
| Maine | KP299751 | [2] | New Hampshire | KP299738 | [2] |
| Maine | KP299752 | [2] | New Hampshire | KP299739 | [2] |
| Maine | KP299753 | [2] | New Hampshire | KP299740 | [2] |
| Maine | KP299754 | [2] | New Hampshire | KP299741 | [2] |
| Maine | KP299755 | [2] | New Jersey | KC801959 | [1] |
| Maine | KP299756 | [2] | New Jersey | KP299745 | [2] |
| Maine | KP299757 | [2] | New Jersey | KP299746 | [2] |
| Maine | KP299758 | [2] | New Jersey | KP299747 | [2] |
| Maine | KP299759 | [2] | New Jersey | KP299748 | [2] |
| Maine | KP299760 | [2] | New Jersey | KP299749 | [2] |
| New York | KP299693 | [2] | New York | KU211527 | [4] |
| New York | KP299694 | [2] | New York | KU211528 | [4] |
| New York | KP299695 | [2] | New York | KU211529 | [4] |
| New York | KP299791 | [2] | New York | KU211530 | [4] |
| New York | KP299792 | [2] | New York | KU211531 | [4] |
| New York | KP299793 | [2] | New York | KU211532 | [4] |
| New York | KP299794 | [2] | New York | KU211533 | [4] |
| New York | KP299795 | [2] | New York | KU211534 | [4] |
| New York | KP299796 | [2] | New York | KU211535 | [4] |
| New York | KP299797 | [2] | New York | KU211536 | [4] |
| New York | KP299798 | [2] | New York | KU211537 | [4] |
| New York | KP299799 | [2] | New York | KU211538 | [4] |
| New York | KP299800 | [2] | New York | KU211539 | [4] |
| New York | KP299801 | [2] | New York | KU211540 | [4] |
| New York | KP299802 | [2] | New York | KU211541 | [4] |
| New York | KP299803 | [2] | New York | KU211542 | [4] |
| New York | KP299804 | [2] | New York | KU211543 | [4] |
| New York | KU211504 | [4] | New York | KU211544 | [4] |
| New York | KU211505 | [4] | New York | KU211545 | [4] |
| New York | KU211506 | [4] | New York | KU211546 | [4] |
| New York | KU211507 | [4] | New York | KU211547 | [4] |
| New York | KU211508 | [4] | New York | KU211548 | [4] |
| New York | KU211509 | [4] | New York | KU211549 | [4] |
| New York | KU211510 | [4] | New York | KU211550 | [4] |
| New York | KU211511 | [4] | New York | KU211551 | [4] |
| New York | KU211512 | [4] | New York | KU211552 | [4] |
| New York | KU211513 | [4] | New York | KU211553 | [4] |
| New York | KU211514 | [4] | New York | KU211554 | [4] |
| New York | KU211515 | [4] | New York | KU211555 | [4] |
| New York | KU211516 | [4] | New York | KU211556 | [4] |
| New York | KU211517 | [4] | New York | KU211557 | [4] |
| New York | KU211518 | [4] | New York | KU211558 | [4] |
| New York | KU211519 | [4] | New York | KU211559 | [4] |
| New York | KU211520 | [4] | New York | KU211560 | [4] |
| New York | KU211521 | [4] | New York | KU211561 | [4] |
| New York | KU211522 | [4] | New York | KU211562 | [4] |
| New York | KU211523 | [4] | New York | KU211563 | [4] |
| New York | KU211524 | [4] | New York | KU211564 | [4] |
| New York | KU211525 | [4] | New York | KU211565 | [4] |
| New York | KU211526 | [4] | New York | KU211566 | [4] |
| New York | KU211567 | [4] | New York | KU211607 | [4] |
| New York | KU211568 | [4] | New York | KU211608 | [4] |
| New York | KU211569 | [4] | New York | KU211609 | [4] |
| New York | KU211570 | [4] | New York | KU211610 | [4] |
| New York | KU211571 | [4] | New York | KU211611 | [4] |
| New York | KU211572 | [4] | New York | KU211612 | [4] |
| New York | KU211573 | [4] | New York | KU211613 | [4] |
| New York | KU211574 | [4] | New York | KU211614 | [4] |
| New York | KU211575 | [4] | New York | KU211615 | [4] |
| New York | KU211576 | [4] | New York | KU211616 | [4] |
| New York | KU211577 | [4] | New York | KU211617 | [4] |
| New York | KU211578 | [4] | New York | KU211618 | [4] |
| New York | KU211579 | [4] | New York | KU211619 | [4] |
| New York | KU211580 | [4] | New York | KU211620 | [4] |
| New York | KU211581 | [4] | New York | KU211621 | [4] |
| New York | KU211582 | [4] | New York | KU211622 | [4] |
| New York | KU211583 | [4] | New York | KU211623 | [4] |
| New York | KU211584 | [4] | New York | KU211624 | [4] |
| New York | KU211585 | [4] | New York | KU211625 | [4] |
| New York | KU211586 | [4] | New York | KU211626 | [4] |
| New York | KU211587 | [4] | New York | KU211627 | [4] |
| New York | KU211588 | [4] | New York | KU211628 | [4] |
| New York | KU211589 | [4] | New York | KU211629 | [4] |
| New York | KU211590 | [4] | New York | KU211630 | [4] |
| New York | KU211591 | [4] | New York | KU211631 | [4] |
| New York | KU211592 | [4] | New York | KU211632 | [4] |
| New York | KU211593 | [4] | New York | KU211633 | [4] |
| New York | KU211594 | [4] | New York | KU211634 | [4] |
| New York | KU211595 | [4] | New York | KU211635 | [4] |
| New York | KU211596 | [4] | New York | KU211636 | [4] |
| New York | KU211597 | [4] | New York | KU211637 | [4] |
| New York | KU211598 | [4] | New York | KU211638 | [4] |
| New York | KU211599 | [4] | New York | KU211639 | [4] |
| New York | KU211600 | [4] | New York | KU211640 | [4] |
| New York | KU211601 | [4] | New York | KU211641 | [4] |
| New York | KU211602 | [4] | New York | KU211642 | [4] |
| New York | KU211603 | [4] | New York | KU211643 | [4] |
| New York | KU211604 | [4] | New York | KU211644 | [4] |
| New York | KU211605 | [4] | New York | KU211645 | [4] |
| New York | KU211606 | [4] | New York | KC801997 | [1] |
| New York | KC801998 | [1] | Texas | OL960648 | [5] |
| Ohio | KC801977 | [1] | Texas | OL960649 | [5] |
| Ontario | MF953375 | [6] | Texas | OL960651 | [5] |
| Ontario | MF953376 | [6] | Texas | OL960652 | [5] |
| Ontario | MF953377 | [6] | Texas | OL960653 | [5] |
| Ontario | MF953378 | [6] | Virginia | KP299785 | [2] |
| Ontario | MF953379 | [6] | Virginia | KP299786 | [2] |
| Ontario | MF953380 | [6] | Virginia | KP299787 | [2] |
| Ontario | MF953381 | [6] | Virginia | KP299788 | [2] |
| Ontario | MF953382 | [6] | Virginia | KP299789 | [2] |
| Ontario | MF953383 | [6] | Virginia | KP299790 | [2] |
| Ontario | MF953384 | [6] | Vermont | KP299728 | [2] |
| Pennsylvania | KC801957 | [1] | Vermont | KP299729 | [2] |
| Pennsylvania | KC801961 | [1] | Vermont | KP299730 | [2] |
| Pennsylvania | KC801962 | [1] | Vermont | KP299731 | [2] |
| Pennsylvania | KC801996 | [1] | West Virginia | KC801950 | [1] |
| South Carolina | KC801975 | [1] | West Virginia | KC801951 | [1] |
| South Carolina | KC801976 | [1] | West Virginia | KC801955 | [1] |
| South Carolina | KC801980 | [1] | West Virginia | KC801970 | [1] |
| South Carolina | KC801981 | [1] | West Virginia | KC801993 | [1] |
| South Carolina | KC801982 | [1] | West Virginia | KC801994 | [1] |
| South Carolina | KC801983 | [1] | West Virginia | KC801995 | [1] |
| South Carolina | KC801984 | [1] | West Virginia | KP299678 | [2] |
| South Carolina | KC801985 | [1] | West Virginia | KP299679 | [2] |
| South Carolina | KC801986 | [1] | West Virginia | KP299680 | [2] |
| South Carolina | KC801987 | [1] | West Virginia | KP299681 | [2] |
| South Carolina | KC801988 | [1] | West Virginia | KP299682 | [2] |
| South Carolina | KC801989 | [1] | West Virginia | KP299683 | [2] |
| South Carolina | KC801990 | [1] | West Virginia | KP299684 | [2] |
| South Carolina | KC801991 | [1] | West Virginia | KP299685 | [2] |
| South Carolina | KC801992 | [1] | West Virginia | KP299686 | [2] |
| South Carolina | KC802003 | [1] | West Virginia | KP299687 | [2] |
| South Carolina | KC802004 | [1] | West Virginia | KP299688 | [2] |
| South Carolina | KC802005 | [1] | West Virginia | KP299689 | [2] |
| Texas | OL960644 | [5] | West Virginia | KP299690 | [2] |
| Texas | OL960645 | [5] | West Virginia | KP299691 | [2] |
| Texas | OL960646 | [5] | West Virginia | KP299692 | [2] |
| Texas | OL960647 | [5] |  |  |  |

# **References**

1. Allison AB, Kevin Keel M, Philips JE, Cartoceti AN, Munk BA, Nemeth NM, Welsh TI, Thomas JM, Crum JM, Lichtenwalner AB, et al. 2014. Avian oncogenesis induced by lymphoproliferative disease virus: A neglected or emerging retroviral pathogen? Virology. 450–451:2–12. doi:10.1016/j.virol.2013.11.037.
2. Thomas JM, Allison AB, Holmes EC, Phillips JE, Bunting EM, Yabsley MJ, Brown JD. 2015. Molecular surveillance for lymphoproliferative disease virus in wild turkeys (*Meleagris gallopavo*) from the Eastern United States. Waldenström J, editor. PLoS One. 10(4):e0122644. doi:10.1371/journal.pone.0122644.
3. Sarid R, Chajut A, Gak E, Kim Y, Hixson CV, Oroszlan S, Tronick SR, Gazit A, Yaniv A. 1994. Genome organization of a biologically active molecular clone of the lymphoproliferative disease virus of turkeys. Virology. 204(2):680–691. doi:10.1006/viro.1994.1584.
4. Alger K. 2015. Lymphoproliferative disease virus (LPDV) in wild turkeys (*Meleagris gallopavo*) in New York State: Diagnostic methods, prevalence, and spatial distribution of a newly discovered pathogen [Master of Science]. [Syracuse, New York]: State University of New York.
5. Cox FJ, Edwards DC. 2021. Tarleton State University. NCBI Genbank Database.
6. MacDonald AM, Jardine CM, Bowman J, Susta L, Nemeth NM. 2019. Detection of lymphoproliferative disease virus in Canada in a survey for viruses in Ontario wild turkeys (*Meleagris gallopavo*). J Wildl Dis. 55(1):113. doi:10.7589/2018-01-013.
