# Supplementary material for: Exploration of lymphoproliferative disease virus strains in wild turkeys (Meleagris gallopavo) in Iowa, USA
Source: PLoS One. 2026 Nov 2;21(11):e0361804. doi: 10.1371/journal.pone.0361804 (PMC13726951; doi:10.1371/journal.pone.0361804)
Supplement: Supplementary file 3 [file pone.0361804.s003.docx]

**S3 Appendix. Phylogenetic relationships of lymphoproliferative disease virus (LPDV) strains (based on sequencing the P31 and a part of the CA (capsid) domains of the virus’s *gag* gene) recovered in Iowa wild turkeys as well as previously catalogued sequences.**

Iowa samples are bolded. Sample names for Iowa turkeys include a unique identifier/State of Iowa/analysis year – County ID.

Sample names from other states and provinces are denoted with their two-letter abbreviation followed by their GenBank accession number (S2 Appendix).
